# Indolethylamine-*N*-methyltransferase Polymorphisms: Genetic and Biochemical Approaches for Study of Endogenous *N,N*,-dimethyltryptamine

**DOI:** 10.3389/fnins.2018.00232

**Published:** 2018-04-23

**Authors:** Jon G. Dean

**Affiliations:** Molecular and Integrative Physiology, Center for Consciousness Science, University of Michigan, Ann Arbor, MI, United States

**Keywords:** indolethylamine-*N*-methyltransferase (INMT), *N,N*-dimethyltryptamine (DMT), consciousness, polymorphisms, psychedelics

## Abstract

*N,N*-dimethyltryptamine (DMT) is a powerful serotonergic psychedelic whose exogenous administration elicits striking psychedelic effects in humans. Studies have identified DMT and analogous compounds (e.g., 5-hydroxy-DMT, 5-methoxy-DMT) alongside of an enzyme capable of synthesizing DMT endogenously from tryptamine, indolethylamine-*N*-methyltransferase (INMT), in human and several other mammalian tissues. Subsequently, multiple hypotheses for the physiological role of endogenous DMT have emerged, from proposed immunomodulatory functions to an emphasis on the overlap between the mental states generated by exogenous DMT and naturally occurring altered states of consciousness; e.g., schizophrenia. However, no clear relationship between endogenous DMT and naturally occurring altered states of consciousness has yet been established from *in vivo* assays of DMT in bodily fluids. The advent of genetic screening has afforded the capability to link alterations in the sequence of specific genes to behavioral and molecular phenotypes via expression of identified single nucleotide polymorphisms (SNPs) in cell and animal models. As SNPs in *INMT* may impact endogenous DMT synthesis and levels via changes in *INMT* expression and/or INMT structure and function, these combined genetic and biochemical approaches circumvent the limitations of assaying DMT in bodily fluids and may augment data from prior *in vitro* and *in vivo* work. Therefore, all reported SNPs in *INMT* were amassed from genetic and biochemical literature and genomic databases to consolidate a blueprint for future studies aimed at elucidating whether DMT plays a physiological role.

## Introduction

The significance of the presence of the powerful psychedelic and serotonergic agonist *N,N*-dimethyltryptamine (DMT) and similar psychedelic compounds in mammalian, including human, bodily fluids remains as unclear as it did when the first such reports surfaced in the 1950s and 60s (Bumpus and Page, 1955; Rodnight, 1956; Franzen and Gross, 1965; Barker et al., 2012). Axelrod (1961) reported an enzyme in rabbit lung that methylated tryptamine to DMT, suggestive of a biological purpose for this pathway and its product. Human and rat brain homogenates incubated with serotonin (5-hydroxytryptamine; 5-HT) and *N*-methyltryptamine were subsequently shown to produce the corresponding dimethylated products (Mandell and Morgan, 1971; Saavedra et al., 1973). The responsible enzyme was named indolethylamine-*N*-methyltransferase (INMT) and has been shown to catalyze the *N*-methylation of a variety of indole compounds via the methyl donor S-adenosyl-L-methionine (AdoMet).

**Figure 1:**
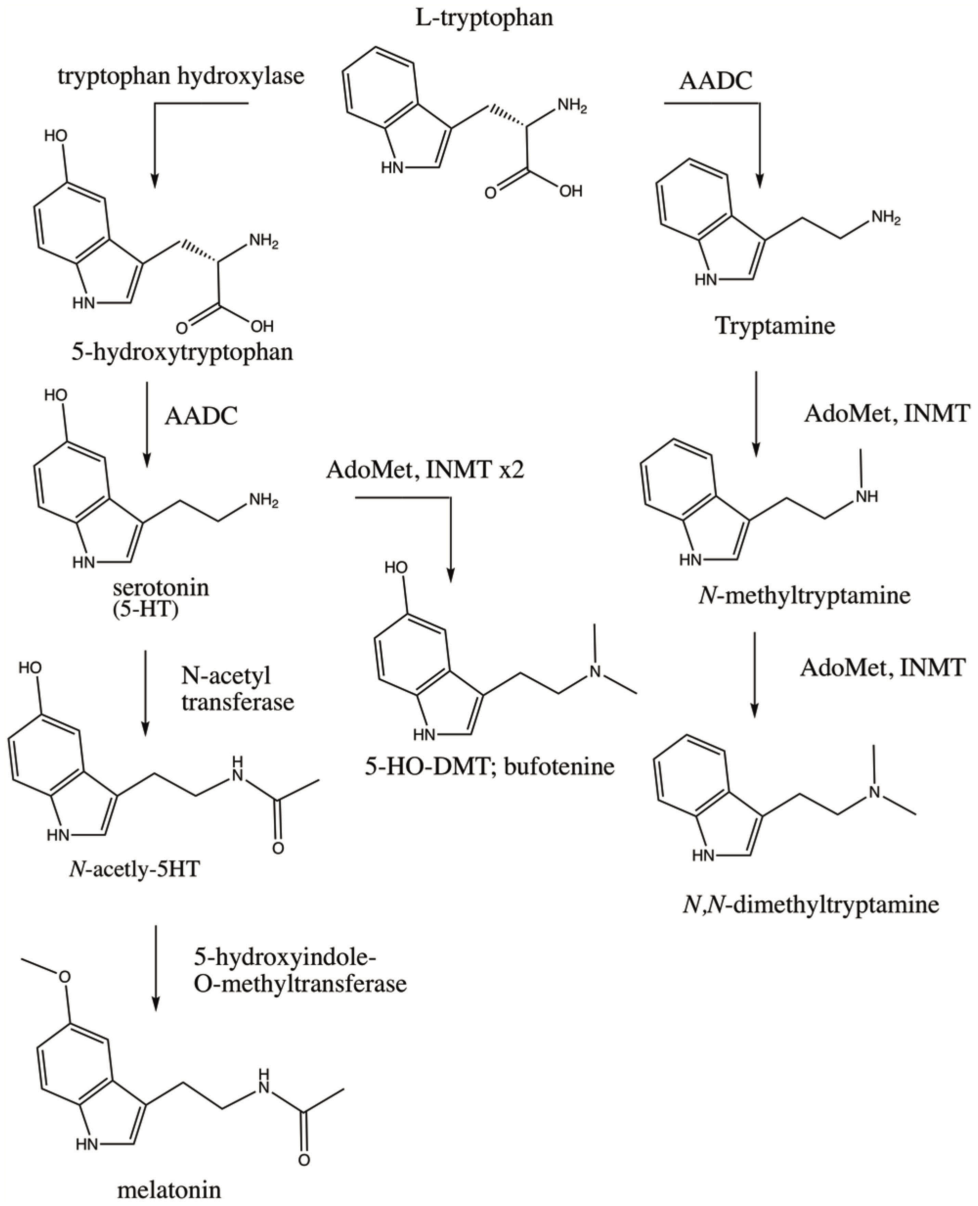
Enzymatic pathways involved in the synthesis of the endogenous DMTs. Dietary tryptophan is the precursor for synthesis of serotonin (5-HT), melatonin, DMT, and 5-HO-DMT (bufotenine). DMT is synthesized via decarboxylation of tryptophan by AADC to produce tryptamine which is then double-methylated utilizing AdoMet as a methyl donor (rightmost pathway). Tryptophan can also be converted to 5-HT via hydroxylation by tryptophan hydroxylase and decarboxylation by AADC (leftmost pathway). 5-HT can be converted to melatonin via addition of an acetyl group by N-acetyl transferase and methylation by 5-hydroxyindole-O-methyltransferase, or can alternatively be converted to bufotenine via methylation by INMT. Polymorphisms in these enzymes, the receptors their products bind to (e.g., 5-HT2A), and/or the metabolic enzymes that degrade their products (e.g., MAO) could affect the levels and effects of the endogenous DMTs through multiple permutations of SNPs as discussed throughout the manuscript.

The finding of *INMT* mRNA in rabbit lung and brain coincided with the cloning of rabbit (Thompson and Weinshilboum, 1998) and subsequently human *INMT* (Thompson et al., 1999). This afforded employment of a battery of modern biochemical assays on purified rabbit and human INMT proteins (referred to hereafter as rINMT and hINMT) that measured the enzyme's activity for methylation of tryptamine and related compounds. Endogenous DMT has since been confirmed in several tissues in humans, rats, and rabbits, including lung and brain (Kärkkäinen et al., 2005). The biosynthesis of DMT first requires decarboxylation of dietary tryptophan via aromatic-*L*-amino acid decarboxylase (AADC) to produce tryptamine, which then undergoes double transmethylation reactions catalyzed by INMT (Figure 1). *INMT* mRNA has been found in several human and rabbit peripheral tissues (Thompson and Weinshilboum, 1998; Thompson et al., 1999), and INMT protein is expressed in the Rhesus macaque pineal gland (Cozzi et al., 2011). An *in vivo* study has further demonstrated the presence of DMT in the dialysate sampled from the pineal gland and visual cortex of freely-moving rats (Barker et al., 2013).

DMT displays affinity for several serotonin and other neurotransmitter receptors (Ray, 2010; Nichols, 2016), exhibits substrate behavior at plasma membrane serotonin transporters in human platelets and rat vesicular monoamine transporters (Cozzi et al., 2009), and accumulates in vesicular fractions following exogenous administration to rats (Barker et al., 1984). Moreover, DMT reportedly crosses the blood brain barrier (Takahashi et al., 1985; Yanai et al., 1986) via active transport. Collectively, these data suggest that DMT may be actively synthesized and released in mammalian tissues, and stored in or transported to the mammalian brain.

Exogenous administration—intravenously, via inhalation, or orally in combination with a monoamine oxidase (MAO) inhibitor—of a pharmacologically active dose of DMT elicits profound alterations in consciousness characterized by visual and other perceptual effects, extreme emotional responses such as euphoria or paranoid ideation, and internally-generated and interactive visual landscapes, accompanied by a sense that the “reality” of what is being perceived is more vivid than dreams or waking reality (Strassman et al., 1994; Gouzoulis-Mayfrank et al., 2005). This, coupled to DMT's close structural similarity to melatonin and serotonin (see Figure 1), has led to several hypotheses of elevated levels of endogenous DMT being involved in naturally occurring altered states of consciousness such as dreaming, religious experience, the near-death state, psychosis, and creativity.

One approach to understanding the possible role of an endogenous hallucinogen was the transmethylation hypothesis (Osmond and Smythies, 1952). Osmond and Smythies (1952) hypothesized that schizophrenia may involve aberrant adrenal metabolism leading to methylation of stress compounds such as adrenaline to form mescaline-like compounds with psychedelic properties that contribute to the clinical symptoms of schizophrenia. This hypothesis has been extended to encompass the possibility of similar altered functioning of DMT enzymatic pathways (see Figure 1) leading to elevated levels of the dimethylated tryptamines (DMTs); e.g., DMT and analog psychoactive compounds such as 5-hydroxy-DMT (5-HO-DMT [bufotenine]) and 5-methoxy-DMT (5-MeO-DMT), in the bodily fluids of schizophrenics (Barker et al., 1981; Grammenos and Barker, 2015). Evolving analytical chemistry-based techniques, from thin layer chromatography to liquid chromatography combined with high resolution mass spectrometry, have been used to compare DMT concentrations in bodily fluids (e.g., blood, urine, cerebrospinal fluid) of psychiatric patients vs. healthy controls. Some of these studies reported a correlation between elevated DMT levels and psychosis, but when considered collectively, ultimately failed to support this hypothesis (Gillin et al., 1976; for a thorough review of all such studies from 1955 to 2010, see Barker et al., 2012). Moreover, upon extrapolation of the concentrations of DMT reported in mammalian bodily fluids and whole tissues to what has been demonstrated via *in vitro* binding assays, it has been hypothesized endogenous DMT concentrations cannot functionally activate brain receptors shown critical for psychedelic effects (Nichols, 2018). This hypothesis is partly based, however, on concentrations observed from exogenous administration and levels observed in the periphery, without consideration for the possibility of direct synthesis and cellular concentration mechanisms for DMT in the brain. Nonetheless, accurate quantitation of DMT concentrations in bodily fluids has been further plagued by its rapid breakdown via MAO (Riba et al., 2015). The first half of this review will focus on summarizing this literature.

These studies demonstrate that research approaches that circumvent the limitations of direct detection of endogenous DMT altogether are needed. Monitoring changes in the activity or a more thorough understanding of the biochemistry of the enzymes involved in DMT synthesis may offer such a solution. One such approach is assessment of INMT activity for synthesis of DMT in association with variation in the *INMT* gene. Variation in the nucleotide sequence of a gene occurs frequently in human populations, and a single individual may have multiple nucleotides that differ from a consensus reference sequence of the gene. These variants are termed single nucleotide polymorphisms (SNPs) when they occur at a single nucleotide and at a notable frequency in a population. Though SNPs do not always manifest at a phenotypic level, they often impact mRNA expression or protein structure via a change in the coding region of a gene, which leads to a change in cellular or behavioral phenotype or even a disease state. Thus, whether or not a SNP has a phenotypic impact relates to the aspect of gene structure that it disrupts. When present in coding regions of the gene, SNPs can either result in (1) no change in the amino acid residue in which it occurs in the translated protein, *a synonymous SNP*, although such a SNP may alter translational or folding events; (2) a change in an amino acid residue, a *nonsynonymous missense SNP*; or (3) the generation of a stop codon in the transcribed mRNA, *a nonsynonymous nonsense SNP*, that often results in a truncated, nonfunctional protein. Additionally, insertions and deletions (indels) of nucleotides can alter the reading frame and subsequently the coding of amino acids that follow its occurrence, often leading to completely nonfunctional protein products being translated. SNPs in non-coding regions can also affect mRNA structure and impact the expression of the gene and the protein it codes through altering the splicing of a gene or binding to DNA of other proteins which regulate expression. The range of impacts a SNP may have can in part be predicted by assessing the SNP's location, the amino acid it changes, or the region it disrupts. This in turn can allow prediction of the level of impact on protein function and expression with relation to a phenotype of interest.

The assessment of SNPs at the single gene and population level is a central tool of genetic studies which have shed light on the biological nature of many disease states. A well-studied example of a disease where a single nucleotide variant in a single gene is responsible is sickle-cell anemia, which results from being homozygous for a SNP (nucleotide A–T) in codon 6 of the beta-globin gene. This leads to a change from the reference amino acid residue glutamic acid (E/Glu) to valine (V/Val) (Ingram, 1959). This elicits aggregation of a sickling hemoglobin in red blood cells in low oxygen environments (Frenette and Atweh, 2007). While the simple Mendelian inheritance pattern of this and other diseases allows for the identification of a single responsible mutation through genetic screens, such as genome wide association studies (GWAS), it is more often the case that mutations in multiple genes associate with complex phenotypes; e.g., bipolar disorder (Serretti and Mandelli, 2008), with each of their individual contributions varying. Coupling such genetic approaches with molecular studies provides a powerful framework to investigate the impact of genomic variation. The potential impact of SNPs in the *INMT* coding region on its enzymatic function, for instance, can be assessed *in vitro* via biochemical assays incubating INMT protein variants with INMT substrates including tryptamine.

As INMT is critical for catalyzing DMT, it can by hypothesized that SNPs leading to differences in the function or levels of INMT may be involved in the manifestation of behavioral phenotypes sharing features with the mental states generated by exogenous DMT. Moreover, identification of *INMT* SNPs in genetic studies not related to psychiatry may also shed light on a non-psychoactive role for endogenous DMT, and such studies will be discussed in the review that follows. Although a number of SNPs in *INMT* have been cataloged, few studies have investigated their occurrence with regard to endogenous DMT synthesis. Therefore, the central goal of this review is to consolidate what is known regarding SNPs in *INMT* to provide a blueprint for future studies on the endogenous DMTs with specific regard to INMT structure and function relationships. This is the focus of the second half of the manuscript, accomplished through review of current genetic and biochemical literature and databases on *INMT*.

## Endogenous DMT and the transmethylation hypothesis

The fact that the subjective effects of exogenous doses of DMT sometimes parallel the positive symptoms of psychosis in schizophrenics—e.g., hyperactivity, delusional thoughts, paranoid ideation, and auditory hallucinations—led to extension of the transmethylation hypothesis to include DMT and INMT (Barker et al., 1981; Cakic et al., 2010; Grammenos and Barker, 2015). Additionally, stress has been shown to both increase DMT levels in rodents (Christian et al., 1977) and exacerbate the positive symptomology of schizophrenia (Lataster et al., 2013). Elevated INMT levels have also been reported in schizophrenics vs. healthy controls (Wyatt et al., 1973a,b).

Thus, there have been attempts by researchers to quantify DMT levels in the bodily fluids of psychiatric populations vs. healthy controls, originally in an effort to develop new drug treatments for schizophrenia that targeted inhibition of INMT. Several of these studies have reported elevated levels of DMT, 5-HO-DMT, and 5-MeO-DMT in the blood, plasma, urine and cerebrospinal fluid of schizophrenics vs. healthy controls (Emanuele et al., 2010; Barker et al., 2012). Many of the studies assessing the DMTs in the bodily fluids of psychiatric patients suffered from antiquated analytical techniques and/or low sample sizes. In the largest review of this literature taking into account all such studies to date, Barker et al. (2012) concluded there was no definitive trend of elevated levels of the DMTs in association with psychosis. Since <1% of the parenteral dose of orally administered DMT is recovered as DMT itself in the urine of volunteers due to its rapid break down by liver MAOs (Riba et al., 2015), it has been suggested that analysis of other DMT metabolites in mammalian bodily fluids is an approach that may prove fruitful (Barker et al., 1981).

Disruption of large-scale cortical synchronization is a correlate of schizophrenia (Uhlhaas and Singer, 2006), and 5-MeO-DMT was found to alter both the firing pattern of rodent medial prefrontal cortex pyramidal neurons as well as decrease cortically generated slow oscillations that underlie the synchronization of global cortical networks. The latter effect was subsequently reversed by the antipsychotic medications clozapine and haloperidol (Riga et al., 2014). Moreover, 5-MeO-DMT is more potent than DMT (McKenna and Towers, 1984; Ott, 1999; Ray, 2010), suggesting endogenous 5-MeO-DMT may be a candidate of focus for future studies aimed at assessing the transmethylation hypothesis. Although the pathway for the biosynthesis of 5-MeO-DMT is not completely clear, INMT has been shown to methylate 5-MeO-tryptamine (Mandel and Walker, 1974). GWAS studies on schizophrenia, however, have yet to identify *INMT*.

Experimental data to date suggest that peripheral endogenous DMT levels are extremely low, and it has been suggested that plasma DMT levels following administration of an exogenous psychedelic dose of DMT are incompatible with what the brain is capable of synthesizing (Nichols, 2018). DMT has been identified in the pineal gland (Barker et al., 2013) of living rats via microdialysis and INMT has also been found in mammalian pineal tissues (Cozzi et al., 2011). But there is not, to date, data quantitating DMT's levels therein or within the brains of any other species via such contemporary analytical techniques. Similarly, studies investigating the possible regional biosynthesis of DMT in the brain are lacking as is research into the possible influence of other physiological factors that could alter its rate of synthesis. It is also inappropriate to assume that the pineal is the sole source of DMT production in the brain. Although it is true that INMT has yet to be reported outside of the pineal gland in mammalian brain, the Northern blot assay employed by Thompson et al. (1999) is not as sensitive as application of INMT antibodies or *INMT in situ* hybridization probes to fixed brain tissues, which may reveal potential cortical or subcortical distributions of INMT protein and *INMT* mRNA.

5-HT2A receptor agonism by psychedelics including DMT has shown to be critical for their psychedelic effects (Vollenweider et al., 1998; Valle et al., 2016), and EC50-values reported for DMT at 5-HT2A receptors are greater than what may be extrapolated from reported peripheral DMT levels in blood (Nichols, 2018). But peripheral levels of DMT may or may not be reflective of brain concentrations, and such samples are from bodily fluids removed from theoretical cellular sources of DMT and thus are further diluted or metabolized. Plasma DMT concentrations observed following administration of a psychedelic dose of DMT may also not mirror synaptic DMT concentrations. For instance, the average concentration of serotonin in brain tissues is in the micromolar range, but synaptic concentrations can reach the millimolar range (Bruns et al., 2000). Moreover, these pharmacological assays that assess DMT activation thresholds at its receptors come with several caveats, the most salient being the shortcomings of removing a receptor from its native environment for subsequent testing. These assays overexpress the protein in a variety of cell culture systems: e.g., HEK-293 cells, human kidney cells which only express 5-HT2A endogenously at low levels; Sf9 insect cells, which do not process proteins or mimic mammalian G-protein coupled receptor signaling pathways; and COS-1 cells, derived from monkey kidney. Overexpression of receptors in these cell systems often leads to artifacts. All are also likely highly mutated from several generations of culturing. This may distance the model from how the receptor functions *in vivo*. However, these are invaluable model systems that allow for control of variables in a manner not possible in intact animals and are intended to be for comparative studies.

Reports of DMT affinity at 5-HT2A have also varied significantly. One study monitoring the formation of [^3^H]-inositol monophosphate formation in fibroblasts expressing 5-HT receptors reported an EC50 of DMT at 5-HT2A to be 983 nM (Smith et al., 1998), while others found this value to be nearly 9-fold lower at 118 nM (Keiser et al., 2009) and 25-fold lower at 38 nM (Blough et al., 2014) via monitoring of calcium response in HEK-293 cells stably expressing 5-HT2A receptors. Here, inter-experimental ratios may be more reliable across several experiments; e.g., DMT's affinity at 5-HT2A vs. 5-HT2C receptors (Smith et al., 1998 observed 983 vs. 49 nM, respectively), than between studies.

Thus, although it is reasonable to suggest that the low amounts of DMT found in peripheral tissues may not occasion a psychedelic response at 5-HT2A and other 5-HT receptors, the ability of DMT to do so, cannot be ruled out based on the current body of literature. As will be discussed, SNPs in *INMT* may alter the endogenous concentrations of DMT, and research on what such concentrations are *in vivo* the intact living mammalian brain where such events occur is needed. With regard to the former, most *in vitro* assays of DMT pharmacology have been conducted on the reference genetic sequences for serotonin receptors, and as such, the currently reported range should not dictate a cutoff EC50-value. One study assayed mutant variants of 5-HT2A and found that non-synonymous SNPs in the coding region of the 5-HT2A receptor increased the potency of tryptamine and decreased that of 5-MeO-DMT, and also increased the affinity and functional effects of several antipsychotic agents *in vitro* (Davies et al., 2006). The authors further concluded that even SNPs predicted to have little impact on 5-HT2A receptor function had unpredictable and substantial effects on its local pharmacology. SNPs in non-coding regions of 5-HT2A associated with schizophrenia (Jönsson et al., 1996) may also alter the function and expression of the protein, and thus impact the interaction of DMT at 5-HT2A or other receptors it competes for. Davies et al. (2006) did not use DMT as a substrate in their studies, so it is unknown the effect the tested SNPs have on DMT's interaction at 5-HT2A receptors.

A one-receptor model of DMT's effects may be overly simplistic and fails to take into account, for instance, radioligand competition assays demonstrating DMT's binding to 5-HT1A, 5-HT1B, 5-HT1D, 5-HT2A, 5-HT2B, 5-HT2C, 5-HT5A, 5-HT6, and 5-HT7 receptors with a range of affinities as low as 39 nM (Keiser et al., 2009). It could be endogenous DMT's interaction at receptors outside of 5-HT2A plays a role in its putative effects. For example, selective 5-HT1A blockade with pindolol magnified subjective responses to DMT by 2- to 3-fold (Strassman, 1996). To suggest schizophrenia is a psychedelic state that requires a threshold amount of endogenous DMT at 5-HT2A also requires understanding dose-response effects for the compound; i.e., non-psychedelic doses of DMT are nevertheless psychoactive (Strassman et al., 1994).

Moreover, adding further complexity to receptor-ligand interactions, it has become widely recognized that a ligand's ability to differentially activate specific cellular second messenger signaling pathways, defined “functional selectivity,” may in part explain why molecularly similar compounds have varying degrees of psychoactivity despite binding the same receptor; i.e., receptors can adopt multiple conformations with which ligands can selectively bind based on their physical properties. With regard to G-protein coupled receptor signal transduction involving 5-HT2A, the most well-known pathway involves biding of a ligand to 5-HT2A to activate G_q_, which induces phospholipase C to hydrolyze phosphatidylinositol, which leads to, amongst other biochemical events, calcium release, and potentially gene transcription. Another less-defined pathway has been identified to occur independently that of the phospholipase C pathway, wherein a ligand can activate phospholipase A_2_ through purported G_12/13_ signaling, which hydrolyzes phospholipids containing arachidonic acid and sets forth a uniquely complex cascade of biochemical events vs. the phospholipase C pathway. 5-HT was shown to have no difference in potency or activity for either the phospholipase C or A_2_ signal transduction pathways, whereas psychedelics including DMT and 5-MeO-DMT have proven more potent for the phospholipase A_2_ pathway. These data suggest the phospholipase A_2_ pathway may be specific to the hallucinogenic effects of psychedelic compounds, although their ability to selectively activate one pathway over the other did not differ (Kurrasch-Orbaugh et al., 2003; Eshleman et al., 2014). Moreover, SNPs in 5-HT2C have shown to impact the ability of agonists to induce specific signaling cascades within these pathways (Berg et al., 2001). The use of human cerebral organoid models in teasing apart which ligands activate which pathways may prove useful, as these systems are made up of heterologous cell clusters capable of differentiating into tissues containing the cellular makeup of broad brain regions. These cultures include multiple neuronal cell types and proteins where DMT interacts including sigma-1 and 5-HT2A/C receptors, and are thus more representative of an *in vivo* neuronal system (Dakic et al., 2017).

Sensitivity to the DMTs may also vary across clinical populations. Although use of psychedelics is generally well-tolerated with judicious regard for dosage and set and setting, there are reports of adverse psychological effects which tend to associate with those harboring preexisting cases of psychosis or nonpsychotic bipolar disorder (Bowers and Swigar, 1983; Hendricks et al., 2015; dos Santos et al., 2017). Davies et al. (2006) have suggested that SNPs in 5-HT2A in their study that lower 5-MeO-DMT's potency may be the genetic basis of such differential response to psychedelics and that individuals who do not bear these mutations and are susceptible to psychosis have an increased likelihood of exacerbation of its development following the use of psychedelics. This may be extended to include a mechanism for how endogenous DMTs play a role in the development of psychosis; that is, certain SNPs in 5-HT2A may increase one's responsiveness to them. It has similarly been proposed that exogenous psychedelics may modulate the activity of INMT and the endogenous DMTs and that this may be intrinsic to their mechanism of action (Barker et al., 1981). As such, SNPs of these kinds may be of interest to proponents of administering psychedelics in clinical settings for amelioration of depression and other ailments, and genetic screening could reveal patients for whom this practice may not be advisable due to their heightened propensity for adverse reactions. SNPs in other genes where DMT interacts may likewise impact its endogenous function. For instance, polymorphisms in the promoter region of the serotonin transporter gene, which may likewise function to remove DMT from the synapse for storage into vesicles (Cozzi et al., 2009), have been linked to depression and increased sensitivity to stress (Caspi et al., 2003) (for a thorough review, see Karg et al., 2011). Further support for this hypothesis is that SNPs in both *AADC* and the serotonin transporter gene (*SLC6A4*) have been associated with depression and anxiety in post-partum populations (Costas et al., 2010).

Aberrant tryptophan metabolism has also been identified in autism, wherein SNPs in *AADC* have been reported (Toma et al., 2013). Deficiencies in melatonin, a hormone produced mainly in the pineal gland in response to the circadian clock signal to provide circadian cues, are prevalent in autism (Melke et al., 2007) as are deficits in rapid eye movement (REM) sleep and overall alterations to the sleep cycle (Limoges et al., 2005; Buckley et al., 2010). Melatonin likewise utilizes tryptophan as a synthetic precursor (see Figure 1), and such lowered melatonin levels could be reflective of shunting of tryptophan toward the synthetic pathway favoring endogenous DMT synthesis. DMT bears a striking chemical resemblance to melatonin (see Figure 1) and has recently been identified in the mammalian pineal gland (Barker et al., 2013) alongside of INMT (Cozzi et al., 2011). 5-HO-DMT has been reported in elevated levels in the urine of autistic individuals (Emanuele et al., 2010), suggesting the unique mental states associated with autism could involve the effects of the endogenous DMTs—although much larger population studies are necessary. The correlations between melatonin pathways, sleep, and autism have been well-studied (Pagan et al., 2014).

In summary, current literature does not show a definitive association of endogenous DMT or *INMT* with schizophrenia and the transmethylation hypothesis. Moreover, it has not been unequivocally demonstrated whether or not endogenous DMT levels ever reach concentrations necessary to activate receptors associated with its psychedelic exogenous effects. However, genetic and biochemical studies on SNPs in *INMT* and its translated protein may provide insight into whether endogenous DMT may be involved in naturally occurring altered states of consciousness or serve other physiological roles, and will be the focus of the remainder of this review.

## Human INMT structure and observed polymorphisms

Human *INMT* is a gene 5,471-bp in length with a reported 792-bp open reading frame that maps to chromosome 7p15.2–p15.3 and encodes a protein 263-amino acids in length that is roughly 29 kDa in mass (Thompson et al., 1999). The ~2.7 Kb *hINMT* mRNA sequence contains 2 alternative exon splice sites that result in translation of two different isoforms of the hINMT protein (isoform 1 = 263 amino acids; NP_006765.4, vs. isoform 2 = 262 amino acids; NP_001186148.1). Though INMT's ability to catalyze DMT from tryptamine is the focus of this article, the enzyme has also been shown to interact with a variety of thioethers and selenium compounds (Mozier et al., 1988; Thompson and Weinshilboum, 1998; Thompson et al., 1999; Chu et al., 2015). A number of nucleotide variants within *INMT* have been identified and will be discussed.

During the initial cloning of *hINMT*, two SNPs resulting in nonsynonymous missense mutations were identified (Thompson et al., 1999) that have since been confirmed to occur at frequencies >10% on the National Center for Biotechnology Information (NCBI) dbSNP database. These particular mutations have subsequently been shown to likely be outside of allosteric and active sites in INMT as will be discussed. Specifically, Thompson et al. (1999) observed that codon 205 could be either methionine (M/Met) or valine (V/Val), and codon 219 could be either glutamic acid (E/Glu) or glycine (G/Gly). The combinations observed were Met205/Glu219, which has since been determined to be the reference combination common to most individuals, and the mutants Val205/Glu219, and Met205/Gly219. Recombinant hINMT proteins expressing the Val205/Glu219 and Met205/Gly219 combinations were created via transfection of COS-1 cells with *hINMT*. Upon subsequent purification, and incubation of hINMT with [^14^C-CH_3_]AdoMet as the methyl donor and tryptamine as the methyl acceptor substrates, the formation of ^14^C-methylated tryptamine was monitored for determination of *K*_m_.

*K*_m_-values provide a logical framework from which to access enzyme-substrate interactions in enzymes obeying Michaelis-Menten kinetics. Interpretation depends highly on pH, temperature, and the rates of the reactions of individual steps making up the whole mechanism. *K*_m_, in essence, is an analysis of how effective an enzyme is at converting substrate to product and corresponds to substrate concentration at half the maximum velocity. Low values reflect a tighter substrate binding when the substrate is rate limiting—although this is not always the case—which infers that a little amount of substrate is enough for the enzyme to run the reaction at half of its maximum velocity.

Thompson et al. (1999) reported that *K*_m_-values for tryptamine were identical for both recombinant mutant proteins (2,920 μM). They then repeated the assays utilizing the rINMT reference Met205/Glu219 (rINMT shares ~90% homology to hINMT, and these two residues are fully conserved), and reported a *K*_m_ for tryptamine an order of magnitude lower at 270 μM vs. the recombinant hINMT mutant variants. They concluded that the higher *K*_m_ for hINMT vs. rINMT for tryptamine in their study suggested tryptamine is not a likely endogenous substrate in humans, an argument echoed by others deeming even a 270 μM *K*_m_ more reflective of tryptamine as a “prototypic substrate” for INMT (Nichols, 2018). However, high *K*_m_-values cannot be taken as evidence against biological activity of a substrate for an enzyme. As can be accessed on GeneCards (http://genecards.org), the *K*_m_ for several enzymes for a given substrate is often orders of magnitude higher than what is likely encountered in the environment of the cell; e.g., chymotrypsin has a *K*_m_ = 5,000–108,000 μM depending on the substrate, and thus this value alone cannot dictate a substrate's biological endogenous activity for the enzyme. Fersht (1999) illustrates that though high *K*_m_-values correspond to lower binding affinity, that catalysis often is carried out at a faster reaction rate and points to glycolytic enzymes that demonstrate catalytic efficiency despite mM affinities. Furthermore, Thompson et al. (1999) drew their conclusion without directly reporting wildtype hINMT (Met205/Glu219) *K*_m_ and comparing it to wildtype rINMT *K*_m_, as it was likely that at this time it was not certain which variant to declare as the reference. The hINMT mutant protein *K*_m_-values were thus also not compared to those of the human wild type protein, so it is unknown whether the interspecies wild type proteins had similar affinities for tryptamine, and further, it is not clear to what degree the mutations in hINMT affected its activity in this study.

Although it is likely that the missense mutations caused a loss of function in hINMT, it cannot be unequivocally declared from comparison of wildtype enzyme activity from one species to mutants of another. Comparing *K*_m_-values across experiments further exposes other caveats. For instance, Chu et al. (2014) reported a *K*_m_ for tryptamine following incubation of INMT from rabbit lung homogenate at 852 μM utilizing assays adapted from Thompson and Weinshilboum (1998), Thompson et al. (1999), results which differ roughly 4-fold vs. what Thompson et al. (1999) reported for rINMT.

Furthermore, a *K*_m_ for tryptamine for purified hINMT wild type protein expressed in *Escherichia coli* has been reported at 850 μM (Chu et al., 2015), which suggests that the mutations observed in Thompson et al.'s (1999) study decreased hINMT's ability to form *N*-methyltryptamine and perhaps DMT. Comparing the results of both Chu studies (2014 vs. 2015) suggests a similar *K*_m_ for rabbit and human INMT, which counters the interpretation of Thompson et al. (1999) that tryptamine may not be an endogenous substrate for hINMT. Moreover, a prior study on methyltransferase activity in human lung preparations reports a *K*_m_-value for tryptamine of 430 μM (Räisänen and Kärkkäinen, 1978). One may consider the activity of the INMT protein in homogenate to be more representative of its function *in vivo*, complete with possible endogenous inhibitors and activators, and the purified protein to counterintuitively be more of an artificial system void of many of the other proteins and substrates that interact with INMT in the whole organism, although the lower *K*_m_ reported for the homogenate could be indicative of the presence of endogenous tryptamine. The INMT proteins isolated from the crude homogenate, however, may also not be the reference protein sequence, further complicating interpretation. Of further note is that this study (Räisänen and Kärkkäinen, 1978) used mass spectrometry to monitor the formation of methylated tryptamines rather than ^14^C-methylated tryptamines.

Future studies could therefore assess the formation of *N*-methyltryptamine or DMT following incubation with tryptamine or *N*-methyltryptamine, rather than incubate with and monitor the formation of non-endogenous radioactive tryptamines. High performance liquid chromatography (HPLC) and/or mass spectrometry can be employed to quantitate these results. This will afford more accurate data better reflective of INMT's function *in vivo*. Future studies assessing the impact of INMT variation should consider these shortcomings when designing experiments to be conducted within the context of the same protocol parameters; e.g., holding constant the same cell systems, substrates, and quantitation. In addition, reports of V_max_, K_m_/V_max_, and K_cat_/K_m_ will also provide greater insight into steps in the reaction pathway. To the latter point, Thompson et al. (2001) and the Chu et al. (2014, 2015) studies have taken such important steps. Despite their caveats, the studies outlined above have provided a valuable blueprint for investigating the biological relevance of mutations in hINMT. This approach offers the potential to observe the molecular implications of clinical findings from genetic screens relevant to the transmethylation hypothesis and other pathophysiological states wherein *INMT* is implicated, as will be discussed.

**Figure 2:**
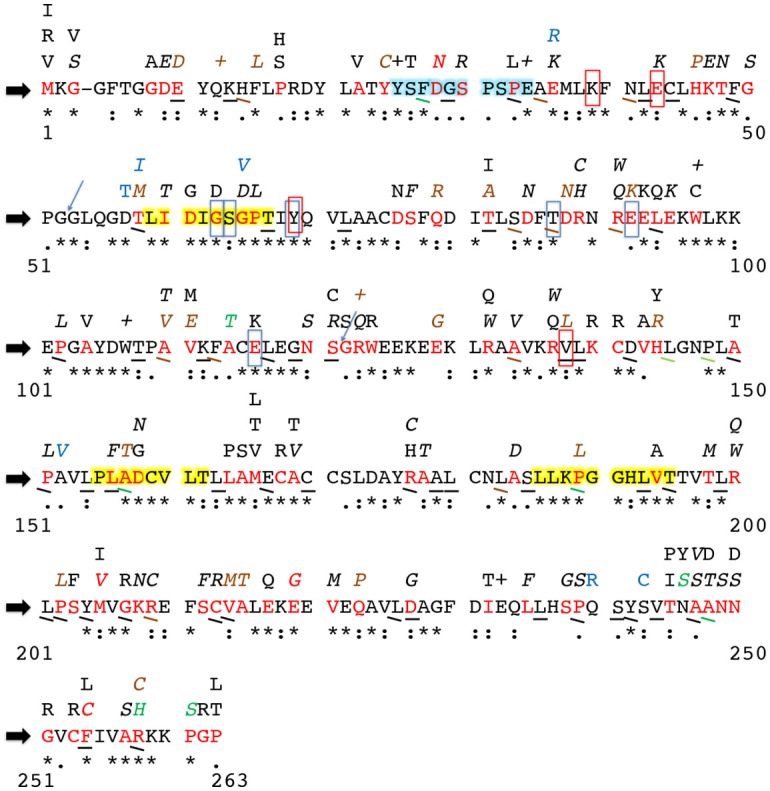
Alterations to the human INMT protein coding region due to single nucleotide polymorphisms. Current literature was consulted to identify amino acid residues in mammalian INMT likely critical to structure/function. The block black arrows at the far left denote the human INMT amino acid reference sequence. Numbers on the far left and right below each row indicate first and last residue numbers in that row. Sequence conservation in human INMT across the mammalian species outlined in Methods is reported underneath each amino acid of the reference sequence as “*”= a fully-conserved residue, “:”= a strongly similar residue, and “.”= a weakly conserved residue. Non-conservation of an amino acid is denoted by absence of these symbols. The amino acids highlighted in yellow are highly conserved regions across the mammalian species compared as well as conserved across methyltransferase enzymes that utilize AdoMet. The amino acids highlighted in light blue are the N-terminal helix-loop-helix region reported to function as an allosteric binding site in hINMT. Residues boxed in red were found to be important to tryptamine binding in rINMT and are conserved in humans; those boxed in light blue represent the same for AdoMet binding. The blue arrows are exon sites. Synonymous SNPs in an amino acid are denoted via underlining of that residue. Missense mutations appear as red amino acid residues in the reference sequence, and the resulting change in this residue is indicated by the letter(s) appearing directly above the red reference residue. A “+” above a reference sequence residue indicates a reported nonsense mutation. Point mutations leading to frame shifts are shown in blue as the resultant altered amino acid residue above the corresponding reference residue. SNPs validated by either multiple independent submissions, the HapMap project and/or the 1000Genome project are denoted by italics or angular underlines. Reported frequencies of each synonymous and nonsynonymous SNP are as follows: black = unknown, brown = <1%, green = 1–10%, red = >10%. No frequencies for frameshift point mutations (blue letters above the reference sequence) were currently reported. Multiple SNPs were often observed at the same reference amino acid residue; e.g., a SNP, in residue 58, threonine (red T), can change the residue to methionine (brown M) at a reported frequency of <1%, whereas a synonymous SNP and a frameshift mutation resulting in a change to isoleucine (blue I) are also reported at unknown frequencies at residue 58. Reported frequencies of SNPs are fluid, and the dbSNP database should be re-examined often to make updates to this figure to match up-to-date literature and subsequent additions to or retractions from the database.

To further such research, the SNPs identified in these studies (and the studies outlined hereafter) as well as SNPs entered into the NCBI dbSNP database were compiled into Figure 2, which shows all currently known SNPs and the amino acid positions at which they occur in the hINMT coding region. The NCBI dbSNP database has amassed SNPs in *INMT* from several sources. Several of the SNPs have been validated by multiple independent submissions, the HapMap project, and/or the 1000Genome project. The database does not remove un-validated SNPs unless user submitters later report it as artifact, as it is often their validation is affirmed through subsequent genotyping. Figure 2 reports all such SNPs, making known those that have been validated. Figure 2 also reports conservation between hINMT and several mammalian species (see Figure 2 legend and section Methods for details). It may be that INMT amino acid residues unique to humans and primates and SNPs therein may be more relevant to the discussion of INMT and DMT with regard to its possible involvement in higher brain activity, whereas amino acid residues fully conserved across lower vertebrates could reflect a different mutual function of INMT that is not involved in such processes.

Chu et al. (2014) hypothesized an allosteric inhibitory binding site for DMT in hINMT based on *in silico* modeling using its crystal structure (Wu et al., 2005) and assays incubating DMT with rabbit lung homogenate. rINMT pre-incubated with 100 μM DMT was found to raise the *K*_m_ for tryptamine from 852 to 1,618 μM. Amino acid residues 25–34 (light blue highlighted region in Figure 2) were confirmed as an N-terminal helix-loop-helix region outside of the enzyme active site where DMT displayed mixed competitive and noncompetitive kinetics. DMT interacts at this site via formation of hydrogen bonds with Asp28 (D) and Glu34 (E) (Chu et al., 2014). As can be seen in Figure 2, a highly frequent human SNP (>10%) in codon 28, which is weakly conserved across mammals, results in a missense mutation to asparagine (N/Asn), whereas a nonsense mutation in codon 34, which is fully conserved across mammals, is reported at an unknown frequency. It was recently proposed that inhibition of INMT by DMT would likely prevent endogenous DMT from ever reaching concentrations necessary to activate its receptors (Nichols, 2018), but it may be that the mutations in these residues results in disruption of this allosteric mechanism, which can be tested *in vitro*.

In another study (Thompson et al., 2001), the AdoMet docking region for rINMT and rat catechol-O-methyltransferase (COMT) were found to be the same via an experimentally-informed computational approach, wherein Tyr69 (Y) (via cation-pi interactions), and Gly63 (G), Ser64 (S), Thr87 (T), Glu92 (E), and Glu116 (E) (vi H-bonding) were all found to facilitate its docking in rINMT (all fully conserved between rINMT and hINMT). Residues important to rINMT-tryptamine binding (fully conserved between rINMT and hINMT unless noted in the following) were deciphered to be Lys39 (K) and Gln200 (Q) (R/Arg in hINMT) (via H-bonding), Glu43 (E), Val138 (V), and Ile202 (I) (P/Pro in hINMT) (via van der Waals interactions), and Tyr69 (Y) (via pi-pi interactions) (Thompson et al., 2001). These residues were compared to hINMT NCBI dbSNP entries to ascertain SNPs in the purported hINMT AdoMet and tryptamine binding sites for entry into Figure 2. The light blue boxes in Figure 2 mark the conserved residues between the reference hINMT and rINMT AdoMet binding site whereas the red boxes mark the same for the tryptamine binding site. As seen in Figure 2, SNPs occur at Glu43 (E), Gly63 (G), Thr87 (T), Glu92 (E), Glu116 (E), and Val138 (V), and may affect INMT affinity for AdoMet and/or tryptamine. hINMT residues Arg200 and Pro202, though not conserved between species, also have observed SNPs that may impact tryptamine binding. Conservation was found between the hINMT and hCOMT AdoMet for Gly63 and Glu116 only, upon alignment with Clustal Omega.

COMT breaks down catecholamines such as dopamine via introduction of a methyl group utilizing AdoMet, and SNPs therein have been implicated in frontal lobe function and risk for schizophrenia (Egan et al., 2001). Certain allele differences have been further noted to impact working memory, and cognitive (Bertolino et al., 2004; Weickert et al., 2004) and dose-responses (Inada et al., 2003) to antipsychotic compounds. Regions of amino acid conservation exist between mammalian COMT and INMT (the first and third yellow-highlighted regions in Figure 2), and one modeling study suggested that the folding of rINMT may be similar to that of rat COMT (Thompson et al., 2001). The first yellow-highlighted region in Figure 2 is conserved across methyltransferases that utilize AdoMet and contains residues important for its binding. Clustal Omega yielded residues of conservation between hINMT and hCOMT (all of these comparisons were made following Clustal Omega alignment of human INMT isoform 1 and human COMT; ascension #: CAG33278.1). Residue Gly63 (of hINMT) is suggested in modulating AdoMet binding in both enzymes, and is conserved. INMT residues Pro189, Leu193, and Thr195 (of hINMT), although not in a binding region, are conserved between the enzymes as well. It may be that these conserved domains inform their functional significance and SNPs seen therein (the yellow regions in Figure 2) may similarly impact INMT's methylating capabilities. A key SNP site reported in schizophrenic studies, Val158Met, negates the ability of COMT to breakdown/methylate dopamine and thus increases the overall amount of dopamine in the prefrontal cortex (Egan et al., 2001; Bertolino et al., 2004; Weickert et al., 2004). This SNP, however, is not conserved or aligned with a region in the INMT reference sequence.

Informed by their model, Thompson et al. (2001) also conducted substrate kinetics on rabbit Val138, centered in the putative active site of the folded INMT protein. Mutation of this residue to tryptophan (W/Trp) increased the *K*_m_ of rabbit wild type INMT from 430 to 3,500 μM, presumably due to introduction of steric hindrance to the active site (Thompson et al., 2001). However, it should be noted no such SNP has yet to be reported in humans (a missense mutation to the only slightly more-bulky amino acid leucine [L/Leu] and a synonymous mutation were entered into dbSNP; see Figure 2). Residue 202 was also predicted to be of importance to the INMT active site in both rabbits and humans in their modeling. This residue, however, differs in rabbits (S/Ser) and (P/Pro) humans. This prompted the authors (Thompson et al., 2001) to mutate rabbit Ser202Pro, which resulted in an increase in *K*_m_ to 1,270 μM. Their interpretation was that Pro202 in humans may account for the reported higher *K*_m_ of hINMT for tryptamine vs. for rINMT, but this study again could benefit from a direct comparison for a wild type *K*_m_ of hINMT for tryptamine for direct intra-study comparison. Table 1 summarizes the main findings of all of the studies discussed in the above section.

**Table 1:** Summary of biochemical assays on INMT from studies reviewed.

**INMT sequence alteration**	**Species**	**Substrate(s) reported**	***K*_m_ (μM)**	**Cell type**	**Publication**
Reference	Human	Tryptamine	850	*E. coli*	Chu et al., 2015
Uncertain	Rabbit	[^14^C-CH_3_]AdoMet + tryptamine	852	Lung homogenate	Chu et al., 2014
Uncertain	Rabbit	[^14^C-CH_3_]AdoMet + tryptamine + DMT	1,618	Lung homogenate	Chu et al., 2014
Reference	Rabbit	[^14^C-CH_3_]AdoMet + tryptamine	430	COS-1	Thompson et al., 2001
Trp138	Rabbit	[^14^C-CH_3_]AdoMet + tryptamine	3,500	COS-1	Thompson et al., 2001
Pro202	Rabbit	[^14^C-CH_3_]AdoMet + tryptamine	1,270	COS-1	Thompson et al., 2001
Reference	Rabbit	[^14^C-CH_3_]AdoMet + tryptamine	270	COS-1	Thompson et al., 1999
Val205/Glu219	Human	[^14^C-CH_3_]AdoMet + tryptamine	2,920	COS-1	Thompson et al., 1999
Met205/Gly219	Human	[^14^C-CH_3_]AdoMet + tryptamine	2,920	COS-1	Thompson et al., 1999
Uncertain	Human	Tryptamine	430	Lung homogenate	Räisänen and Kärkkäinen, 1978

## *INMT* polymorphisms identified in genetic screens

Recent genetic studies have indicated SNPs in *hINMT* that likely affect function in relation to certain phenotypes/populations. Following patient genotyping, His46Pro, Pro188Leu, Gly218Glu, and Cys253Phe were found to be SNPs in the coding region of hINMT associated with the intestinal disease, Hirschsprung's—defined by absence of ganglion cells in the colon—in Korean subjects (Kim et al., 2015). It should be noted that the numbering on all but the His46Pro SNP are exclusive to hINMT isoform 2 (NP_001186148.1), whereas isoform 1 (NP_006765.4) was used for generation of Figure 2. Isoform 2 is shortened by 1 residue (262 vs. 263; amino acid Gly52 is absent in isoform 2), and it may be that specific splice isoforms of hINMT associate with certain phenotypes such as Hirschsprung's disease (these 3 SNPs are referenced as Pro189, Glu219, and Phe254 on isoform 1 on dbSNP and in Figure 2; it should further be noted that reference and SNP residues in positions 218 and 253 are reversed in Kim et al. (2015) vs. their inputting on dbSNP; i.e., they are Glu218Gly and Phe253Cys on the latter database, and Figure 2 reflects the dbSNP indexing at isoform 1 on codons 219 and 254). SNPs were also found in the non-coding region of INMT including the promoter region (6 total), intronic region (3 total), and the 3′-untranslated region (2 total), which could feasibly affect mRNA transcription and translation of INMT. The authors performed *in silico* analysis of His46Pro, which is not highly conserved among mammals (see Figure 2), to determine that this mutation is likely to cause damage to the protein. The association of INMT with Hirschsprung's may be related to the documented absence of serotonergic neurons in the gut in those with the disease (Rogawski et al., 1978). Kim et al. (2015) concluded that a lowered amount of serotonin in relation to His46Pro may lead to lower efficiency of enteric neural crest migration, the most well-documented cause of the disease.

INMT regulates the N-methylation of tryptamines, and tryptophan is the required precursor for both tryptamine and serotonin biosynthesis. Tryptophan can either be converted to DMT via the previously mentioned AADC-INMT pathway, or to 5-hydroxy-L-tryptophan via tryptophan hydroxylase and then to serotonin following decarboxylation via AADC. Moreover, serotonin can be methylated by INMT to form 5-HO-DMT (bufotenine), a compound with anecdotal psychedelic properties that reportedly exhibits higher affinity for 5-HT2A than DMT (Ray, 2010; Shen et al., 2010), although serotonin activity at the enzyme is lower than for tryptamine (Mandell and Morgan, 1971; Thompson and Weinshilboum, 1998). These pathways are outlined in Figure 1. In light of this, the finding of INMT SNPs linked to an intestinal disease like Hirschsprung's becomes intriguing when coupled to a report of DMT and 5-HO-DMT in human stool (Kärkkäinen et al., 2005). This could suggest a peripheral regulatory role for DMT and/or 5-HO-DMT in gastrointestinal function, as serotonin has a well-characterized role therein. A recent study also found an association between selective serotonin uptake inhibitor use and increased risk of a child developing Hirschsprung's (Nielsen et al., 2017), adding more data to a recent line of inquisition into a gut-brain link in association with depressive states. Indeed, 95% of all serotonin in the body is found in the gut, despite its known criticality for maintaining homeostatic brain physiology.

The enteric nervous system contains several 5-HT receptor subtypes within families 5-HT1–5-HT7 expressed on multiple cell types; e.g., enteric neurons, and enterochromaffin cells within epithelial mucosa that are located in close proximity with immune cells also known to express 5-HT receptors. 5-HT mediates several aspects of enteric physiology; e.g., peristalsis, and colonic contraction, as well as responses to pathophysiological events through interaction at these sites (Hasler, 2009; Manocha and Khan, 2012). Hirschprung's is not only characterized by abnormal enteric development, but inflammation is also a commonly observed complication of the disease (Frykman and Short, 2012). Agonism at the 5-HT2B receptor, where DMT has displayed moderate affinity (Blair et al., 1999; Keiser et al., 2009), appears to be critical for proper enteric development (Gershon and Ratcliffe, 2004) and activation of 5-HT1 and 5-HT2 receptors by endogenous psychedelics such as DMT and 5-HO-DMT may significantly interfere with the intracellular cascade within immune cells to lead to suppression of the production of inflammatory cytokines (Szabo, 2015). This effect has been demonstrated following induction of inflammation in human primary monocyte-derived dendritic cells pretreated with 100 μM of DMT (Szabo et al., 2014). Similar involvement of DMT in inflammation pathways was reported by Dakic et al. (2017), who tested broad changes in the human neural proteome following 5-MeO-DMT application utilizing human cerebral organoids. They reported several differences in protein expression between vehicle (0.3% ethanol) and treatment group (24-h incubation with 5-MeO-DMT) via mass spectrometry. However, the 13 μM dose utilized is much greater than what a brain would naturally encounter. Such a system nonetheless represents a step in the direction of determining not only an endogenous role for the DMTs, but also the concentrations that may be necessary to induce such molecular changes. The impact of lower doses of the DMTs in the same or similar protocols could elucidate this. The *INMT* upregulation observed in Hirschprung's thus suggests a feasible physiological role for the DMT's in proper enteric function worthy of future investigation.

Another recent GWAS conducted on poor metabolizers of selenium, a substrate methylated by INMT, showed SNPs in *INMT* to significantly associate with blood and urinary trimethylselenonium ion concentrations, a common selenium metabolite (Kuehnelt et al., 2015). The top SNPs were Phe254Cys (F254C in Figure 2, a coding region high frequency SNP in a highly conserved residue), and two lead SNPs in the 3′ untranslated region of *INMT* in complete linkage disequilibrium; i.e., non-random association indicating conserved mutual inheritance. These SNPs were indexed as rs6970396 (in an enhancer region) and rs1061644 (in an open chromatin region), and do not appear in Figure 2 as they are outside the coding region. Of the Bangladesh women sampled, those with reference/wild type rs6970396 (nucleotide reference G instead of base A) had lower levels of the trimethylselenonium ion metabolite. Levels of this metabolite were also associated with a dosage effect of this allele; i.e., low levels were found in those homozygous for the reference (nucleotides GG), and increasingly higher levels for heterozygous and homozygous base A, respectively (i.e., nucleotides AG < AA for increase in the trimethylselenonium ion metabolite) were observed. Because INMT methylates selenium compounds, inheriting the A allele leads to a gain of function of this mechanism or its occurrence, and theoretically the same for the methylation of tryptamine to form DMT. The A allele was found to result in gain of two transcription factor binding sites (GATA1, GATA2) and loss of 2 others (PEA3, XBP1). XBP1, one of the lost transcription factors associated with inheriting the A allele, is linked to immune/inflammatory responses (Iwakoshi et al., 2003) and cellular endoplasmic reticulum stress response (Yoshida et al., 2006)—two functions recently proposed for DMT (Frecska et al., 2013; Szabo et al., 2016).

Moreover, the Phe254Cys SNP (nucleotide T to G; also referenced rs4720015) was closely situated to rs6970396, in complete linkage disequilibrium with both 3′ SNPs, and associated with lower levels of the trimethylselenonium ion vs. the reference Phe254. Despite not being at the active site, which is strongly conserved among mammals, this residue was predicted to affect INMT protein structure and/or function (via http://sift.jcvi.org and http://genetics.bwh.harvard.edu/pph2/). The ancestral T allele in rs1061644 (vs. the C allele) was also associated with high concentrations of the trimethylselenonium ion as well as with a gain of two binding sites (TFAP2A, Enktf1) and a loss of three others (cJun, ATF3, ER-alpha). Thus, variation in the non-coding region of *INMT* was associated with either a gain of methylation activity or enzymatic expression, or a combination of both, for INMT as assessed in relation to the above alleles via urinary trimethylselenonium ion concentrations using HPLC and inductively coupled plasma mass spectrometry. Of further interest to the 3 SNP sites reported in the selenium GWAS study is that the lowest frequencies for rs6970396 (nucleotide A), rs1061644 (nucleotide T), and wild type rs4720015 (Phe254/nucleotide T) alleles were reported for the Argentinian Andean (1.5%) and Peruvian (5%) cohorts. Thus, these populations appear to have the least active INMT methyltransferase activity (for selenium) of the several populations investigated. This is of interest because the DMT-containing brew ayahuasca has been pervasive in the indigenous cultures of South America for centuries (McKenna, 2004; McKenna and Riba, 2018). It is not necessarily the case that selenium binds to the same docking sites on the INMT enzyme as tryptamine. But INMT overall expression is likely lower in comparison to wild type in these populations, and it can therefore be inferred that lower levels of the endogenous DMTs may be observed as well. These allelic variations are however more than likely linked to dietary differences in these populations with regard to INMT's methylation of selenium.

**Figure 3:**
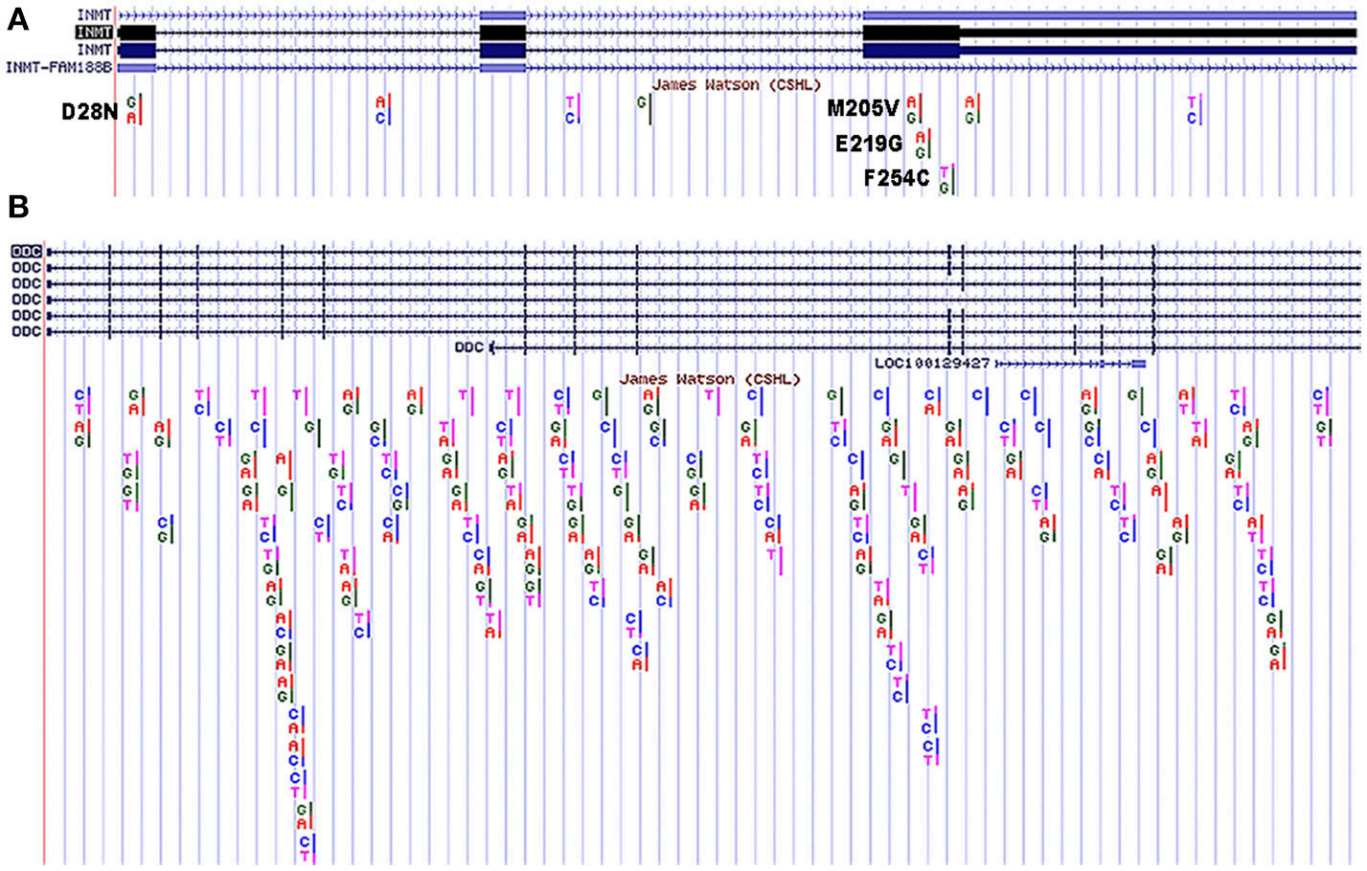
SNPs in James Watson's *INMT* isoform 1 and *DDC* isoform 2 from UCSC Genome Browser. **(A)** SNPs found in James Watson's *INMT* isoform 1. Coding region SNPs are in black and seen in exons 1 and 3 as noted: D28N, M205V, E219G, and F254C. These SNPs are discussed in detail in the text. The *INMT* sequence highlighted black reflects isoform 1. **(B)** SNPs found in James Watson's *DDC* isoform 2. The *DDC* sequence highlighted blue at the top of the figure reflects isoform 2. The two genes are not shown comparatively to scale.

The selenium study (Kuehnelt et al., 2015) illustrates how multiple SNPs can be inherited that collectively may impact the methylating capacity of INMT. To further illustrate this complexity, the public access genome of molecular biology pioneer James Watson was analyzed for SNPs in his *INMT* coding region that may affect INMT protein function. Using the UCSC Genome Browser (GRCh37/hg19), four such SNPs in James Watsons's *INMT* (isoform 1) that led to missense mutations were found. These were: D28N, M205V, E219G, and F254C (Figure 3A). As can be seen in Figure 2, D28N is in the allosteric loop wherein DMT binds to inhibit INMT. The relevance of these SNPs in concert with regard to protein function remains unknown, but can be assayed, and each of these SNPs has been specifically discussed throughout this manuscript. The UCSC Genome Browser was also used to align *DDC* (another nomenclature for *AADC*) isoform 2 to the Watson genome (Figure 3B), as SNPs leading to alterations in AADC protein expression or function may be pertinent to INMT function and the mechanism of DMT synthesis (see Figure 1). There have been reports of enhancement of creativity and divergent thinking following the use of psychedelics (Harman et al., 1966; Sessa, 2008; Baggott, 2015) including ayahausca (Frecska et al., 2012; Kuypers et al., 2016). From Kekulé's closed-eye visions of tetravalent whirling snakes leading to the discovery of the structure of benzene, to an ethereal hairpin melody from Brian Wilson's paracusia, the question arises, “Can endogenous psychedelics, such as DMT, aid in facilitating the egression of exceptional thought-processes from the mundane?”

Piechota et al. (2012) used a whole-genome microarray profiling technique to assess temporal transcriptional alterations in mice during 12 days of administration of methamphetamine or heroin and following a 12-day withdrawal period. Among the 27 genes identified, they found *INMT* mRNA expression was gradually enriched in the rodent nucleus accumbens of basal forebrain and to a lesser degree the dorsal striatum during administration of both compounds. The highest expression was in the heroin group and observed on day 6, and levels descended to basal activity during withdrawal. The authors concluded that *INMT* up-regulation and subsequent increase in DMT synthesis may be responsible for the psychotropic effects of drug-withdrawal. However, recent data shows that human administration of psychedelics structurally related to and including DMT can ameliorate the symptoms of addiction (Grob et al., 1996; Johnson et al., 2014; Mithoefer et al., 2016), as well as comorbid disease states such as depression (Osório et al., 2015; Carhart-Harris et al., 2016, 2017) in which self-medicating is a hallmark (Markou et al., 1998), wherein the degree of the positive therapeutic outcome correlates with the magnitude of the peak acute psychological effects (Roseman et al., 2018). A recent study has shown a decrease in retinal and occipital cortex neuronal response to color contrast in depressed individuals (Bubl et al., 2015) suggesting a scientific basis for the often-experienced “lack of color” in this illness. INMT has been found in the monkey retina (Cozzi et al., 2011), and underproduction of a molecule such as DMT—known to evoke heightened perception of color—may be one molecular mechanism behind such changes in perception in depression. Moreover, application of high doses of 5-MeO-DMT to human neuronal organoid cultures led to up-regulation of integrins, which is also observed in depressive patients that respond well to antidepressants (Dakic et al., 2017). DMT has also been proposed to serve an anxiolytic role via its binding at the trace amine receptor (Jacob and Presti, 2005), which would not necessitate endogenous levels reaching those of a psychedelic dose.

Dakic et al. (2017) also identified a down-regulation of metabotropic glutamate receptor 5, and knockout of this protein or its antagonism in mice leads to attenuation of addictive behaviors (Dakic et al., 2017). Thus, the upregulation of *INMT* observed by Piechota et al. (2012) may be reflective of increased endogenous DMT synthesis following the addictive behavior, although DMT was not tested in the proteomics study. Chronic administration of methamphetamine in rodents also induces up-regulation of sigma-1 receptor mRNA (Stefanski et al., 2004). DMT is a sigma-1 receptor agonist (Fontanilla et al., 2009), and agonism at this receptor has been shown to ameliorate depressive behavior in rodents (Moriguchi et al., 2014). Conversely, sigma-1 knockout mice display depressive behaviors; e.g., immobility in the forced swim test (Sabino et al., 2009), although such a conclusion drawn from this data may be erroneously interpretating the loss of a hyperlocomotion response, which is known to be mediated in part via sigma-1 receptor agonism (Fontanilla et al., 2009), as depression. Methamphetamine also binds to sigma-1 receptors and it may be this interaction is responsible for their up-regulation in addiction. However, sigma-1 knockout mice still exhibit hyperlocomotion when administered methamphetamine whereas they no longer respond in this manner to DMT (Fontanilla et al., 2009). INMT is also localized to postsynaptic sites of C-terminals of rodent motor neurons in close proximity to sigma-1 receptors (Mavlyutov et al., 2012), perhaps providing an anatomical foundation to a proposed role of INMT and DMT in regulating the hypermobility response associated with addiction. However, that sigma-1 receptors are so ubiquitously expressed throughout mammalian tissues and are chaperone proteins integral to calcium signaling suggests this colocalization may not be of unique significance to motor neurons. It may also simply be that the up-regulation of *INMT* observed by Piechota et al. (2012) reflected INMT's reported *N*-methylation of xenobiotics for their degradation.

## Conclusion

This review has focused on assessing prior studies on endogenous DMT employing analytical chemistry and pharmacological assays and amassing what is known regarding variation in the INMT enzyme from the results of genetic- and biochemical-based studies. Figure 2 represents a summary of the latter data. It also suggests potential solutions to current shortcomings of approaches toward understanding whether there is a physiological role for the endogenous DMTs. Current literature employing population genetics and gene expression-based protocols supports a possible role of the DMT-synthesizing *INMT* gene in drug addiction. Less support exists with respect to psychosis viz. the transmethylation hypothesis, as GWAS studies have yet to associate *INMT* with such phenotypes. Furthermore, it is likely that if the endogenous DMTs are involved in conscious experience—normal or abnormal—that several receptors, proteins, and enzymes that regulate and/or utilize tryptophan and tryptamine will function at different efficacies in different individuals in association with such phenotypes as influenced by their genetic code. Epigenetics has also shown that gene expression can be influenced by environment, experience, and learning, though these discussions are beyond the scope of this review.

Figure 1 outlines some of the different enzymes and substrates in the endogenous DMT pathways discussed throughout this article. Combined with Figure 2, the complexity of the molecular biology potentially associated with subjective experience and behavioral phenotypes points to the limitations of such associative data. Furthermore, although *INMT* SNPs identified in genetic screens of populations with a phenotype of interest can be assessed using *in vitro* models, such models function outside of the organismal level and should also be interpreted with this in mind. Although SNPs identified by GWAS represent codons of interest with potential to change gene and protein function when identified in a gene such as *INMT*, they are not always causal. Depending on the nature of these studies, they may rather indicate that the causal SNP is nearby. A powerful approach recently implemented in *in vitro* work is to culture cells from actual patients/populations presenting with phenotypes/SNPs of interest. This model may be employed to elucidate the molecular mechanisms of INMT and endogenous DMT and represents an important compliment to synthetic approaches utilizing recombinant INMT proteins (such as those outlined in Table 1).

Understanding how the presence of multiple SNPs in *INMT* affects INMT protein expression and function with regard to DMT synthesis is required to elucidate the function of endogenous DMT (as outlined in Figure 3A). Particular SNPs in *INMT* may be found to alter the levels of endogenous DMT in different populations. It may be that the same patients/populations similarly present with SNPs in the coding or non-coding regions of DMT receptors that alter the sensitivity to endogenous DMT. Though studies combining computational and biochemical approaches have been undertaken (Davies et al., 2006; Keiser et al., 2009), compuational power has vastly increased in the interim since their publication. Combining these expanded genetic and biochemical protocols will be valuable to the study of the endogenous DMTs with regard to normal and abnormal mental states as well as other proposed physiological roles for DMT such as mediating aspects of inflammation and immunomodulation.

## Methods

### Generation of INMT polymorphism Figure 2

INMT amino acid coding region sequences were obtained from the NCBI database for: human isoform 1 (NP_006765.4), chimpanzee (JAA26545.1), monkey (NP_001253042.1), rabbit (AAC97491.1), mouse (EDK98723.1), rat (NP_001102492.1), pig (NP_001231662.1), and cow (NP_001179577.1). These sequences were entered into Clustal Omega for sequence alignment. Conservation was reported as “^*^” = a fully-conserved residue, “:” = a strongly similar residue, and “.” = a weakly conserved residue. Next, all known frequencies (at the time of the generation of this figure) of reported synonymous, nonsynonymous, and frameshift-causing SNPs in human INMT (geneID: 11185) were determined using NCBI's dbSNP database (plus strand; NP_006765.4; SNP GeneView reports GRCh38) and entered into the figure as described in the Figure 2 legend. Figure 2 was then adapted to also reflect known literature on INMT SNPs conserved and deemed important for active and allosteric site function as well as SNPs suspected to be deleterious to proper protein folding (Thompson et al., 1999, 2001; Wu et al., 2005; Chu et al., 2014; Kim et al., 2015; Kuehnelt et al., 2015). These SNPs were entered into the figure as applicable and are described in detail in the Figure 2 legend.

## Author contributions

The author confirms being the sole contributor of this work and approved it for publication.

### Conflict of interest statement

The author declares the absence of any commercial or financial relationships that could be construed as a potential conflict of interest.
